# Preliminary findings indicate nosocomial transmission and Roma population as most affected group in ongoing measles B3 genotype outbreak in Bulgaria, March to August 2017

**DOI:** 10.2807/1560-7917.ES.2017.22.36.30611

**Published:** 2017-09-07

**Authors:** Anna Kurchatova, Stefka Krumova, Nadezhda Vladimirova, Lubomira Nikolaeva-Glomb, Asya Stoyanova, Todor Kantardjiev, Nina Gatcheva

**Affiliations:** 1 National Centre of Infectious and Parasitic Diseases, Sofia, Bulgaria; 2 Bulgarian Association for Prevention and Infection Control – BulNoso, NGO, Sofia, Bulgaria

**Keywords:** Bulgaria, viral infections, measles, infection control, measles-mumps-rubella (MMR) vaccine, outbreaks, surveillance, vaccines and immunisation, epidemiology, molecular methods

## Abstract

From March to August 2017, 165 measles cases were reported from three regions in Bulgaria. The age range was 0−55 years and 66% of the cases were under 9 years. The Roma population was disproportionally affected (89% of cases), 41% cases were unvaccinated and in 24 cases there was nosocomial transmission mostly in paediatric departments. A child under 12 months of age died. Control measures have been taken and the investigation is still ongoing.

## Outbreak description

Since 17 March and until 31 August 2017, a total of 204 suspected measles cases were reported to the web-based National Measles Surveillance System (NMSS) in Bulgaria. In Bulgaria, measles has been a statutorily notifiable disease since 1921 [1]. National case-based notification started in 2004 and in 2005, the European Union (EU) case definition and case classification were adopted for surveillance purposes [2,3]. The NMSS was developed and introduced in 2009 (http://mmr.gateway.bg/en/).

Here we present preliminary findings and the control measures implemented at the beginning of the ongoing measles outbreak.

### Case definition and classification

For the outbreak investigation, the EU measles case definition is being used [2]. Any person meeting the clinical criteria i.e. fever, maculopapular rash and at least one of cough/coryza/conjunctivitis, is classified as possible case; any person meeting the clinical criteria and with an epidemiological link to a laboratory-confirmed case is classified as a probable case; and any person not vaccinated in the previous 6 weeks and meeting the clinical and the laboratory criteria is defined as a confirmed case.

Thirty-nine cases did not fulfil the case definition and were discarded on the basis of laboratory criteria and epidemiological data [4].The remaining 165 cases were classified according to [2], as follows: 122 confirmed, 36 probable and seven possible cases. All cases were epidemiologically investigated and laboratory tests were performed at the National Reference Laboratory for Measles, Mumps and Rubella (NRL-MMR) at the National Centre of Infectious and Parasitic Diseases (NCIPD) in Sofia. Sera collected in the region of Plovdiv after mid-May were tested for IgM/IgG at the Medical University of Plovdiv.

### Epidemiology and case characteristics

**Figure 1:**
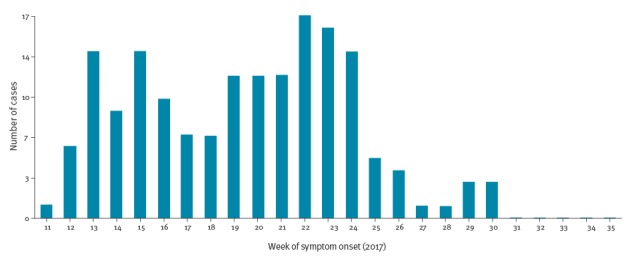
Measles cases by week of symptom onset, Bulgaria, March−August 2017 (n = 165)

The index case in the outbreak was a 2-year-old child from a large Roma community in Plovdiv. The child was admitted to the infectious diseases ward of the regional hospital with diarrhoea and other enterocolitis symptoms. After 3 days, the child developed typical measles symptoms and the national health authorities were informed on the same day. There was further transmission in the family or in the hospital setting. The epidemic curve of cases by week of symptom onset shows the dynamics of outbreak (Figure 1).

Males (n = 93) were more affected than females (n = 72; ratio: 1:0.8). The majority of cases (n = 107; 65%) were children under 9 years of age. The median age of cases was 4 years (range: < 1−55). A high number of cases were not immunised (n = 67, 41%) and nearly half of them (n = 35) were children under 1 year of age who are not eligible for vaccination.

Among vaccinated cases, those having received at least one dose of a measles-containing vaccine (77; 47%) were prevailing. The distribution of measles cases by age groups and vaccination status is shown in Table 1.

**Table 1:** Distribution of measles cases by age groups and vaccination status, Bulgaria, March−August 2017 (n = 165)

Vaccination status (number of doses administered)	Age group (years)							
	< 1	1–4	5–9	10–14	15–19	20–29	> 30	Total
0	35	20	2	0	0	5	5	67
1	0	27	21	5	4	2	1	60
2 or more	0	0	1	7	7	2	0	17
Unknown	1	0	0	0	2	4	14	21
**Total**	**36**	**47**	**24**	**12**	**13**	**13**	**20**	**165**

Complications were registered in nine cases: two presented with pneumonia and seven with diarrhoea. One of the confirmed measles cases, a child under 1 year of age, died.

**Figure 2:**
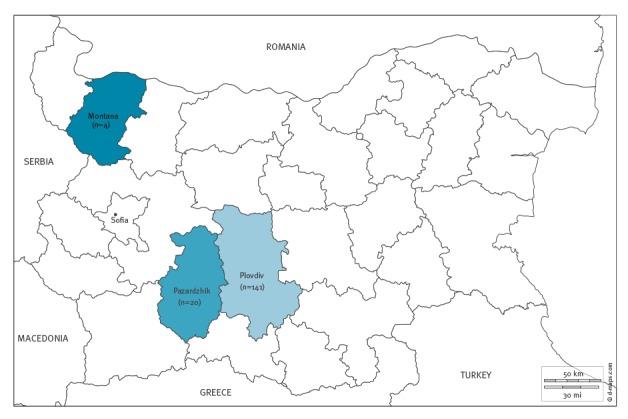
Measles cases by region, Bulgaria, March−August 2017 (n = 165)

Cases were recorded in three of the 28 Bulgarian administrative regions (Figure 2). As in the previous large measles outbreak in Bulgaria (2009–2011), the Roma population living in Plovdiv and Pazardjik was the most affected group (n = 147 cases, 89%). The cases described here represent the three clusters from the regions of Plovdiv, Pazardjik, and Montana. The epidemiological link between Plovdiv and Pazardjik clusters is under investigation.

Map obtained from d-maps: http://d-maps.com/carte.php?num_car=5666&lang=en.

#### Plovdiv region

The index case was found here on 17 March 2017. This is the most affected region with 141 (85%) measles cases reported. Among these cases, 100 (71%) are confirmed, 35 (25%) are probable and six (4%) are possible cases. Almost all measles cases (127, 90%) were registered among the Roma community. The median age of cases is 5 years (range: < 1–55).

#### Pazardjik region

The first case was reported to the NMSS on 13 May 2017, in a child aged 15 months, vaccinated at the end of March 2017 with the first dose of the measles-mumps-rubella vaccine (MMR1). A total of 20 cases were reported to the NMSS from this region: 18 confirmed at the NRL-MMR, one probable and one possible. All measles cases in this region were occurred in Roma population with a median age of 2.5 years (range: < 1−21).

#### Montana region

The first case was reported on 2 May 2017 in a non-vaccinated adult in their late 20s, who had visited Romania three weeks before symptom onset, where there is a large measles outbreak ongoing with 8,937 cases recorded between 1 January 2016 and 25 August 2017 [5]. The following case occurred in a family member. Another two sporadic cases were also registered in this region. A total of four cases were reported, all of them confirmed at the NRL-MMR.

## Laboratory confirmation

From 17 March to 31 August 2017, a total of 79 measles cases were tested at the NRL-MMR with a commercial indirect enzyme-linked immunosorbent assay (anti-Measles IgM/IgG antibodies, Euroimmun test kit, Luebeck, Germany). All samples collected from measles cases received at the NRL were tested for IgM and IgG antibodies against both measles and rubella. The remaining samples were tested for anti-measles IgM/IgG antibodies in regional laboratories.

Viral RNA extraction and amplification were performed for 41 urine and 56 nasopharyngeal specimens using QIAGEN kit (QIAGEN GmbH, Hilden, Germany).

**Figure 3:**
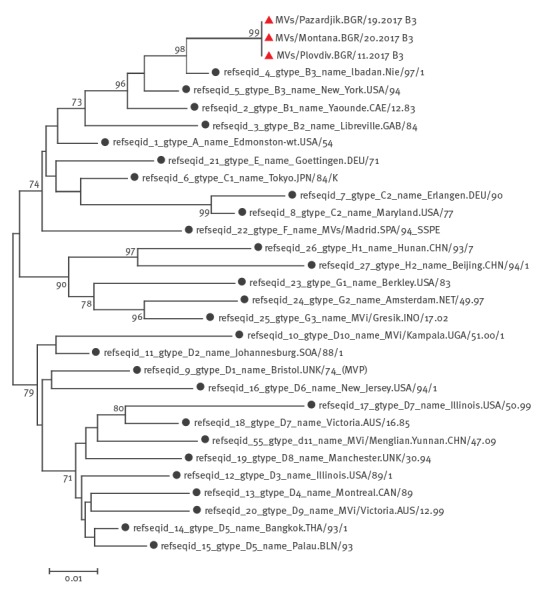
Phylogenetic tree showing the measles genotype B3 in Bulgaria, March to August 2017

Genotyping and phylogenetic analysis were performed at the World Health Organization (WHO) European Regional Reference Laboratory for Measles and Rubella, Robert Koch Institute, Berlin, Germany and at the NCIPD in Sofia. Sequential analysis of the samples of the initial cases in Plovdiv, Pazardjik and Montana showed measles genotype B3, which has been dominantly circulating in Europe (France, Italy and Romania) over the past 2 years (Figure 3).

Based on C-terminal 450 nucleotides of the Nucleoprotein (N) gene of the measles virus and World Health Organization reference strains [17].The phylogenetic tree was generated by MEGA version 5.05 by neighbour-joining method with 1,000 bootstrap replicates; only bootstrap values of ≥ 70 are shown. Sequences from Bulgaria (n = 3) are marked with a red triangles.

## Control measures

Following the risk assessment of the situation related to increasing measles incidence in many European countries and a permanent threat of importation [6], the Bulgarian Ministry of Health (MoH) disseminated an official warning letter to all Regional Health Inspectorates (RHI) on 13 March 2017.

A National Coordination Council for the management and response to the measles outbreak was established ad hoc at the MoH on 22 March 2017, after the detection of the outbreak. Letters with recommended control measures were provided immediately: general practitioners were requested to check the immunisation status of all persons aged between 13 months and 18 years and to immunise all those unvaccinated with one dose of MMR vaccine; medical specialists in hospitals and outpatient settings were requested to be alert to the early detection, timely reporting, isolation, diagnosis, and treatment of measles cases. It was also recommended to hospitalise patients with measles living in crowded households, in order to ensure better conditions for treatment and care and to minimise the spread of the disease in communities with poor living conditions. Therefore, most of the cases in the affected regions were admitted to the infectious diseases ward (159, 96%).

## Vaccination coverage in Bulgaria

The measles monovalent vaccine was introduced in Bulgaria in 1969. Consecutively, the combined MMR vaccine was implemented for the first dose given at 13 months of age (in 1993) and the second dose at 12 years (in 2001). The analysis of the past 5 years reveals that vaccination coverage with the two doses of MMR is sub-optimal (< 95%) (Table 2). A downward trend of the MMR vaccine coverage, mostly expressed for the second MMR dose, has been observed. During the past 3 years, the national average was below 90%. Sub-national analysis shows that one third of the regions have reported an MMR1 coverage below 90%.

**Table 2:** Measles-mumps-rubella vaccination coverage at national and sub-national level, Bulgaria, 2012–2016

Year	MMR1 coverage (%)	Regions with < 90% coverage / total regions	MMR2 coverage (%)	Regions with < 90% coverage / total regions
**2012**	93.7	3/28	94.0	3/28
**2013**	95.1	5/28	93.5	4/28
**2014**	93.2	5/28	**88.6**	14/28
**2015**	91.5	9/28	**86.9**	16/28
**2016**	92.1	9/28	**88.3**	15/28

MMR: measles-mumps-rubella.

## Discussion

Following the occurrence of the index case in March 2017, three clusters of measles cases were registered in Bulgaria. Roma population living in two neighbouring regions in Plovdiv and Pazardjik was the most affected group.

According to the latest national census in Bulgaria in 2011 [7], the population consists of Bulgarians (84.8%), Turks (8.8%) and Roma (4.9%). Roma inhabit all regions of the country. They live in overcrowded communities often under poor sanitary conditions and they are characterised by frequent travel within the country and abroad [8].

Two main features of the ongoing outbreak can be outlined: (i) the existence of a vulnerable group of Roma population non-immunised or insufficiently immunised and (ii) nosocomial measles transmission in the paediatric departments of two hospitals in Plovdiv. The recommendation to hospitalise vulnerable Roma children, may also have contributed to the latter.

The analysis of the immunisation status of the cases shows a prevalence of those having received at least one dose of a measles-containing vaccine (77; 47%) however, some of them might have been immunised during their incubation period and thus, these data need to be interpreted with caution.

The last indigenous measles cases in the country before the outbreak in 2009–2011 were registered in 2001. Since then, several importations were reported [9,10]. The measles case that occurred in 2009 was followed by a nationwide outbreak with 24,364 cases (cumulative incidence for April 2009−December 2011: 326/100,000 inhabitants) and a resurgence of endemic measles in the country [11]. Along with the sub-optimal national vaccination coverage with two doses of MMR vaccine registered during the past 5 years, the current outbreak revealed that pockets of non-immunised and insufficiently immunised population, especially in Roma communities, continue to exist.

The low vaccination coverage is a risk factor for importation and spread of measles in Bulgaria. Continued strengthening of surveillance and improving vaccination coverage in general population is need to avoid the spread of the outbreak to other regions of the country and to prevent exportation. The current outbreak also shows that special intensified vaccination activities should be directed to the Roma population.

Measles outbreaks affecting vulnerable groups, including Roma communities, have been reported in other European countries [12-16].
